# The Pathology and Treatment of Sexual Impotence

**Published:** 1899-08

**Authors:** 


### Books and Magazines.

THE PATHOLOGY AND TREATMENT OF SEXUAL IMPOTENCE. By Victor G. Vecki, M. D., San Francisco, California. From the Author's Second German Edition. Revised and Rewritten; pp. 291; price, $2.00, net. Philadelphia: W. B. Saunders, 925 Walnut Street, 1899.

The first edition of this book appeared ten years ago, and created at the time some unfavorable comment from the portion of the profession generally known as "back numbers." The subject has always been important, though no one had turned the modern calcium upon it and rescued it from superstition and quackery, where antiquity and the church had left it. It required ability and courage to investigate the subject and promulgate the truths and theories which resulted therefrom. The modern profession is to be congratulated upon the timely appearing of Vecki's book, and it is predicted that this edition will find more earnest readers than the two former editions.

The author discusses the subject under the following heads:

The introduction, Anatomy; Physiology of the Sexual Act; Etiology of Impotence; Forms of Impotence; Diagnosis; Prognosis; Prophylaxis; Treatment and Special Therapeutics.

It is a valuable book and well worth the serious consideration of the professional as well as the lay reader. T. J. B.

